# Cell type‐specific transcriptomics of esophageal adenocarcinoma as a scalable alternative for single cell transcriptomics

**DOI:** 10.1002/1878-0261.12680

**Published:** 2020-04-21

**Authors:** Max Krämer, Patrick S. Plum, Oscar Velazquez Camacho, Kat Folz‐Donahue, Martin Thelen, Isabel Garcia‐Marquez, Christina Wölwer, Sören Büsker, Jana Wittig, Marek Franitza, Janine Altmüller, Heike Löser, Hans Schlößer, Reinhard Büttner, Wolfgang Schröder, Christiane J. Bruns, Hakan Alakus, Alexander Quaas, Seung‐Hun Chon, Axel M. Hillmer

**Affiliations:** ^1^ Institute of Pathology Faculty of Medicine and University Hospital Cologne University of Cologne Germany; ^2^ Department of General, Visceral and Cancer Surgery Faculty of Medicine and University Hospital Cologne University of Cologne Germany; ^3^ FACS & Imaging Core Facility Max Planck Institute for Biology of Ageing Cologne Germany; ^4^ Center for Molecular Medicine Cologne University of Cologne Germany; ^5^ Cologne Center for Genomics University of Cologne Germany; ^6^ Cancer Therapeutics and Stratified Oncology Genome Institute of Singapore Agency for Science, Technology and Research (A*STAR) Singapore City Singapore

**Keywords:** cancer‐associated fibroblasts, cell types, esophageal adenocarcinoma, transcriptomics, tumor microenvironment

## Abstract

Single‐cell transcriptomics have revolutionized our understanding of the cell composition of tumors and allowed us to identify new subtypes of cells. Despite rapid technological advancements, single‐cell analysis remains resource‐intense hampering the scalability that is required to profile a sufficient number of samples for clinical associations. Therefore, more scalable approaches are needed to understand the contribution of individual cell types to the development and treatment response of solid tumors such as esophageal adenocarcinoma where comprehensive genomic studies have only led to a small number of targeted therapies. Due to the limited treatment options and late diagnosis, esophageal adenocarcinoma has a poor prognosis. Understanding the interaction between and dysfunction of individual cell populations provides an opportunity for the development of new interventions. In an attempt to address the technological and clinical needs, we developed a protocol for the separation of esophageal carcinoma tissue into leukocytes (CD45+), epithelial cells (EpCAM+), and fibroblasts (two out of PDGFRα, CD90, anti‐fibroblast) by fluorescence‐activated cell sorting and subsequent RNA sequencing. We confirm successful separation of the three cell populations by mapping their transcriptomic profiles to reference cell lineage expression data. Gene‐level analysis further supports the isolation of individual cell populations with high expression of *CD3, CD4, CD8, CD19*, and *CD20* for leukocytes, *CDH1* and *MUC1* for epithelial cells, and *FAP, SMA, COL1A1,* and *COL3A1* for fibroblasts. As a proof of concept, we profiled tumor samples of nine patients and explored expression differences in the three cell populations between tumor and normal tissue. Interestingly, we found that angiogenesis‐related genes were upregulated in fibroblasts isolated from tumors compared with normal tissue. Overall, we suggest our protocol as a complementary and more scalable approach compared with single‐cell RNA sequencing to investigate associations between clinical parameters and transcriptomic alterations of specific cell populations in esophageal adenocarcinoma.

## Graphical abstract

Little is known about the interactions between the different intratumoral cell types, that is, epithelial tumor cells, cancer‐associated fibroblasts, and immune cells. We developed a scalable and cost‐effective workflow to separate these cell types from esophageal adenocarcinoma (EAC) biopsies using fluorescence‐activated cell sorting and subsequent RNA sequencing. Using this approach, we characterize these cell types in EAC and normal tissue.

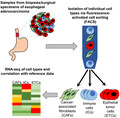

**Abbreviations**
CAF: cancer‐associated fibroblast
DEG: differentially expressed gene
DMSO: dimethyl sulfoxide
EAC: esophageal adenocarcinoma
EMT: epithelial‐to‐mesenchymal transition
FACS: fluorescence‐activated cell sorting
FAP: fibroblast activation protein
FBS: fetal bovine serum
FDR: false discovery rate
FITC: fluorescein isothiocyanate
GO: gene ontology
LD: life dead
MMP: matrix metalloproteinase
NSCLC: non‐small cell lung cancer
PCA: principal component analysis
PDGF: platelet‐derived growth factor
PDGFRα: PDGF receptor α
PD‐L1: programmed death ligand 1
RCA: reference component analysis
scRNA‐seq: single‐cell RNA sequencing
TGF‐β: transforming growth factor β
VEGF: vascular endothelial growth factor

## 1 Introduction

Esophageal cancer is associated with the sixth‐highest mortality rate of cancer‐related deaths and presumed to be one of the most mortal malignancies worldwide (Coleman *et al.*, 2018). Over the past decades, there has been a rapid increase particularly in the incidence of esophageal adenocarcinoma (EAC) in the Western world, but despite improvements in perioperative treatments, there is no sufficient therapeutic strategy for the majority of patients so far (Coleman *et al.*, 2018). Further, prediction of therapy success is poor and response to neoadjuvant therapies varies dramatically ranging from no to complete response (Vallböhmer *et al.*, 2010).

Focus in cancer research has shifted from considering epithelial cancer cells to analyses of their interactions with different components of the tumor microenvironment. Particularly, the immune cell population has revealed an immense effect on tumor progression. Today, immune checkpoint blockage is first‐line therapy in several solid neoplasia such as malignant melanoma and non‐small cell lung cancer (NSCLC) (Incorvaia *et al.*, 2019; Spagnolo *et al.*, 2019). For EAC, several biomarkers in the immune compartment have been detected, and currently, clinical trials are ongoing to evaluate the safety and efficacy of possible immunotherapies (Tanaka *et al.*, 2017).

There is also rising evidence for an important impact of cancer‐associated fibroblasts (CAFs) on tumor biology and disease progression (Shiga *et al.*, 2015). CAFs are a heterogeneous cell population of unknown origin that form the stromal part of solid tumors (Shiga *et al.*, 2015). They release cytokines (e.g., TGF‐β), proteases (e.g., matrix metalloproteinases), and growth factors (e.g., VEGF, PDGF) with various autocrine and paracrine functions that can enhance tumor growth, neovascularization, and migration of cancer cells (Kakarla *et al.*, 2012). In several cancer types, including colorectal, breast, ovarian, and head and neck cancer, the presence of CAFs correlates with poor prognosis (Lai *et al.*, 2012; Marsh *et al.*, 2011; Tsujino *et al.*, 2007; Yamashita *et al.*, 2012). In esophageal cancer, Wang et al. described pleiotropic functions in carcinogenesis, proliferation, angiogenesis, and metastasis (Wang *et al.*, 2016). Although CAFs obviously influence tumor biology, they are still poorly characterized. Since targeting either CAFs or their secreted paracrine factors could improve therapeutic response, characterization of the roles of fibroblasts in EAC can help developing clinically effective treatments (Kakarla *et al.*, 2012).

Single‐cell transcriptome sequencing (scRNA‐seq) has opened new avenues for the understanding of the biological role of cell populations, their origins, and interactions (Ren *et al.*, 2018). Single‐cell approaches – in contrast to bulk tissue sequencing – allow to determine which cell type is responsible for transcriptomic changes, which is of fundamental importance for the understanding of tumor biology. Single‐cell RNA sequencing of colorectal cancers, for example, has shown that epithelial‐to‐mesenchymal transition (EMT) signature genes are upregulated in CAFs and not in epithelial cells (Li *et al.*, 2017), a phenomenon that might have gotten misinterpreted as EMT by bulk sequencing. Hence, a systematic analysis of gene expression profiles in different tumor cell types of EAC in comparison with normal esophageal mucosa will help to understand the role of the interaction between (cancer associated) fibroblasts, immune cells, and epithelial tumor cells in carcinogenesis and disease progression. Single‐cell sequencing, however, is still expensive, limiting the number of clinical samples that are usually profiled per study. More scalable approaches for the analysis of individual cell populations of larger series of tumors are warranted to be able to identify cell type‐specific alterations that are associated with clinical features. It is therefore desirable to develop economic methodologies that allow to analyze cell types separately but not necessarily on a single‐cell level.

To address this need, we developed a protocol to isolate different cell types (epithelial cells, immune cells, fibroblasts) of endoscopically obtained EAC tissue as well as normal esophageal mucosa and to sequence the transcriptome of each cell type separately. By mapping the transcriptomes to reference tissue expression data, we demonstrate the successful separation of the three cell types. Further, we explored the utility of this approach by comparing expression profiles of esophageal normal mucosa with adenocarcinoma tissues. Our protocol provides a scalable tool for the systematic and cost‐effective investigation of the individual cell populations of EAC. It will help to improve our understanding of pathological processes and possibly identify novel therapeutic targets in EAC.

## 2 Methods

### 2.1 Patients and tumor samples

Fresh tissue samples from nine patients with histologically confirmed adenocarcinoma of the distal esophagus or the gastroesophageal junction were prospectively collected between June and December 2018. In six of these patients, both corresponding tumor and normal esophageal mucosa were taken, while in one patient only tumor tissue and in two patients normal mucosa had been biopsied. Processing and consecutive separation of these samples led to 31 cell populations after FACS sorting with sufficient yield for further analysis via RNA‐seq.

All patients underwent primary staging including esophagogastroduodenoscopy, endoscopic ultrasound, and spiral contrast‐enhanced computer tomography of thorax and abdomen within the Department of General, Visceral and Cancer Surgery at the University Hospital of Cologne. Written informed consent was obtained from all patients before participating in the analysis, and the study was approved by the local Institutional Review Board (Ethics No. 18‐274). The study's methodologies conformed to the standards set by the Declaration of Helsinki and its later amendments.

Samples were taken either by endoscopic biopsy (six samples) or obtained from surgical specimens (three samples). During endoscopy, standardized biopsies from both tumor and corresponding normal esophageal mucosa at 5‐cm distance were taken and immediately transferred into 1 mL of RPMI 1640 medium (Thermo Fisher Scientific, Waltham, MA, USA) at room temperature for further processing.

Standard surgical procedure was laparotomic or laparoscopic gastrolysis and right transthoracic en bloc esophagectomy including two‐field lymphadenectomy of mediastinal and abdominal lymph nodes (Ivor Lewis esophagectomy) as described previously (Plum *et al.*, 2018). Samples obtained from a surgical specimen were also immediately transferred into RPMI 1640 medium at room temperature and further processed as described below.

In case of neoadjuvant treatment (five patients), either neoadjuvant chemoradiation analog CROSS (four patients) or perioperative chemotherapy analog FLOT regimen (one patient) was applied (Al‐Batran *et al.*, 2016; van Hagen *et al.*, 2012).

### 2.2 Single‐cell dissection

**Fig. 1:**
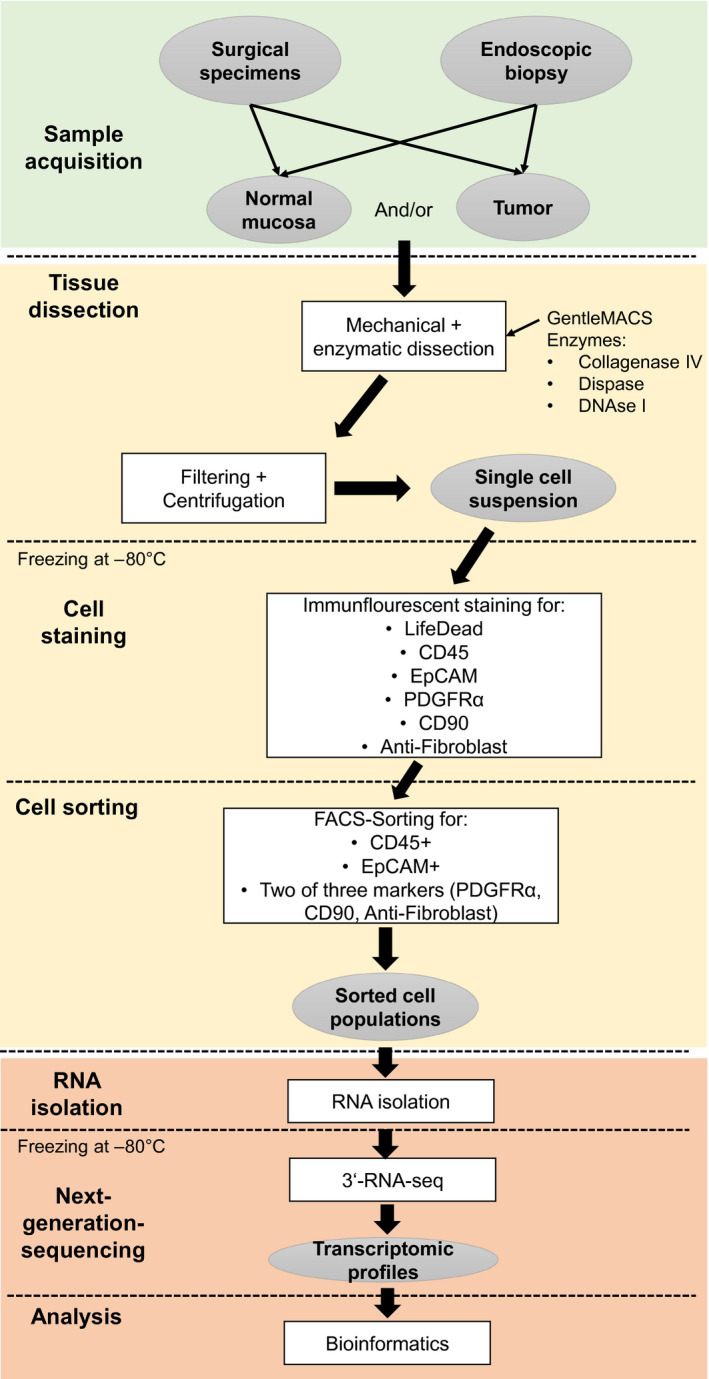
Schematic representation of the workflow.

Immediately after endoscopy, corresponding tumor and normal mucosa biopsies were processed separately (Fig. 1). Tissue samples were transferred together with 1 mL of Gibco™ RPMI 1640 Medium (Thermo Fisher Scientific) and 1 mL of PBS (Thermo Fisher Scientific) into Petri dishes and dissected mechanically using two scalpels. After transfer into gentleMACS C Tubes (Miltenyi Biotec, Bergisch Gladbach, Germany), additional 1 mL of each of the following enzymes was added: DNAse I (500 U·mL^−1^; AppliChem PanReac, Darmstadt, Germany; in PBS), collagenase IV (320 U·mL^−1^; Thermo Fisher Scientific; in PBS), and dispase II (2 U·mL^−1^; Sigma‐Aldrich, St. Louis, MO, USA; in PBS). Automated tissue dissociation was performed using the preset human tumor programs 1, 2, and 3 of the gentleMACS™ Dissociator (Miltenyi Biotec). Mechanical dissociation steps 1 and 2 were followed by enzymatic digestion for 20 min while rotating at 37 °C. Following enzymatic‐mechanical tissue dissociation, samples were diluted with PBS and filtered through a Falcon^®^ 100‐µm cell strainer (Corning, New York, NY, USA) to remove larger debris. Filtering was repeated if necessary. Dissociated cells were collected by centrifugation at 405 ***g*** for 5 min at room temperature. Cells were resuspended by vortexing within freezing medium including 60% Gibco™ RPMI 1640 medium (Thermo Fisher Scientific), 30% FBS (Capricorn Scientific, Ebsdorfergrund, Germany), and 10% dimethyl sulfoxide (DMSO) (Sigma‐Aldrich). Afterward, cells were frozen at −80 °C for 24 h and then transferred to liquid nitrogen for long‐term storage until fluorescence‐activated cell sorting (FACS). As one freeze/thaw cycle of the single‐cell solutions resulted in hemolysis, no additional erythrocyte lysis was performed.

### 2.3 Immunofluorescence staining of single‐cell suspension

Frozen single‐cell suspensions were thawed, added to 10 mL of Gibco™ RPMI 1640 Medium (Thermo Fisher Scientific) at 37 °C and vortexed. Before further processing, 500 µL of each sample was separated and mixed with 1 mL of medium for the unstained control. Samples were centrifuged at 450 ***g*** for 5 min at room temperature. After centrifugation, all supernatants were discarded. The collected cells were resuspended in 500 µL MACS buffer [PBS (pH 7.2) + 2 mm EDTA + 0.5% BSA] and kept on ice until FACS analysis. The following incubation steps were performed on ice in the dark. Samples were stained consecutively with the following monoclonal anti‐human antibodies: 2 µL Alexa Fluor^®^ 647‐conjugated anti‐PDGF receptor α (PDGFRα; Cell Signaling Technology, Danvers, MA, USA) and 1 µL eBioscience™ Fixable Viability Dye eFluor™ 506 (Thermo Fisher Scientific) for 15 min followed by 5 min of incubation with 1 µL PE/Cy7‐conjugated anti‐CD45 (Biolegend, San Diego, CA, USA). Cells were incubated for 10 min with additional 2 µL FITC‐conjugated anti‐EpCAM (Miltenyi Biotec), 5 µL PE‐conjugated anti‐fibroblast (Miltenyi Biotec), and 2 µL VioBlue‐conjugated anti‐CD90 (Miltenyi Biotec). Additional staining for epithelial cells utilizing 1 µL APC/Fire™ 750‐conjugated anti‐mouse/human CD324 (E‐Cadherin) (Biolegend) was performed in six samples. This additional staining was omitted in subsequent samples as E‐Cadherin did not stain additional epithelial cells that were not stained by EpCAM, including normal esophagus (data not shown). Cells were spun down at 450 ***g*** for 5 min at 4 °C. Supernatants were discarded and collected cells resuspended in 500 µL cold MACS buffer.

Simultaneously to the cells, compensation beads were prepared for analysis by flow cytometry utilizing the ArC™ Amine Reactive Compensation Bead Kit for life‐dead (LD) staining (Thermo Fisher Scientific) and the AbC™ Total Antibody Compensation Bead Kit (Thermo Fisher Scientific), respectively, according to the manufacturer's instructions. Immunofluorescent stained cell suspensions and beads were kept on ice until sorting.

### 2.4 Flow cytometry analysis and sorting

Sorting of the single‐cell suspensions was performed using a BD FACSAria Fusion (BD Biosciences, San Jose, CA, USA) using a 100‐µm nozzle and 20 psi pressure, using aerosol containment. Immediately before analysis, cell suspensions were filtered once again using a 70‐µm CellTrics strainer (Sysmex, Kobe, Japan). Gating strategy was as follows: After viability gating, cells were gated according to the surface expression of CD45 as marker for immune cells (‘immune cell population’). CD45‐negative cells were analyzed for the expression of PDGFRα, fibroblast marker, and CD90. Those cells which were positive for at least two of those markers were defined as fibroblasts (‘fibroblast cell population’). Finally, all other CD45‐negative cells were analyzed for expression of EpCAM (or E‐Cadherin) as marker for tumor cells of epithelial origin (‘epithelial cell population’). Cell subpopulations were sorted into 500 µL cold MACS buffer at 4 °C.

### 2.5 RNA isolation and next‐generation sequencing

After sorting, cells were kept on ice and RNA isolation was performed using the PicoPure™ RNA Isolation Kit (Thermo Fisher Scientific) according to manufacturer's instructions. Isolated RNA was stored at −80 °C. Libraries for RNA sequencing were prepared using the QuantSeq 3′ mRNA‐Seq Library Prep Kit FWD for Illumina (Lexogen GmbH, Vienna, Austria) according to the low‐input protocol. Libraries were sequenced on a HiSeq 4000 (Illumina) by 1× 50 bases.

### 2.6 RNA‐seq analysis

Reads were aligned to the human genome (*Homo sapiens* GRCh38) using star software v. 2.6 (Dobin *et al.*, 2013). Mapped reads were counted with HTSeq, and differential gene expression analysis was conducted using bioconductor r package deseq2 version 1.22.2. (Love *et al.*, 2014). An adjusted *P*‐value threshold of 0.05 and a log2 fold change ≥ 1 were set to determine differential gene expression. The complete lists of DEGs in a pairwise manner are available in the Table S1.

### 2.7 Reference component analysis

Clustering of the independent samples was performed using the r package reference component analysis (rca) v. 1.0. (Li *et al.*, 2017) with the default option ‘Global Panel’, and this panel contains a set of featured genes from the reference bulk transcriptomes in the HumanU133A/GNF1H Gene Atlas and the Primary Cell Atlas. The resulting RCA clusters for the different samples were plotted as heat maps or principal component analysis (PCA) as part of the downstream analysis pipeline of the rca package.

### 2.8 Pseudo‐bulk analysis

Processed RNA‐seq gene counts for all the analyzed cells were obtained from the supplementary datasets in Owen *et al. *(2018). Single‐cell datasets from four patients were selected (A, D, E, and F), where A and D are patients with Barrett's esophagus and E and F are normal esophageal mucosae of patients without Barrett's esophagus. For each of the four analyzed patients, a sample of 100 single cells was randomly selected and the processed counts from each patient were aggregated by obtaining the mean for each gene across the 100 sampled cells to emulate a pseudo‐bulk dataset. The resulting averaged counts were used as input to perform the RCA.

### 2.9 Gene ontology

After differential gene expression analysis was performed among the different flow cytometry‐sorted cell populations, genes whose expression was exclusively upregulated in comparison with the remaining cell types were analyzed with the go software (http://geneontology.org/; Ashburner *et al.*, 2000; Carbon *et al.*, 2019) to test for over‐representation of common biological processes using all genes as background. To investigate tumor/normal differential expression, all expressed genes of the respective cell type defined by the sum of normalized expression tag counts > 1 were used as background.

## 3 Results

### 3.1 Patients' characteristics

Tumor samples from nine patients were collected including seven male and two female patients. Median age of all patients participating was 65 years (minimum: 57 years, maximum: 83 years). Initial tumor grading was G2 in five patients and G3 in two patients, pT stage was pT1 in one patient, pT2 in three patients, pT3 in two patients, and pT4 in one patient (no information available for 2 patients since those patients did not undergo surgery due to metastasis). Four patients received neoadjuvant chemoradiation analog CROSS, and one patient received perioperative chemotherapy analog FLOT regimen. Full baseline characteristics are provided in Table 1.

**Table 1:** Baseline characteristics of patient included.

Variable	Total (*n* = 9)	
	Number (*n*)	Percentage
Age		
Median (min–max)	65 years (57–83 years)	
Gender		
Male	7	77.8
Female	2	22.2
Anatomical localization		
Esophagogastric junction	8	88.9
Gastric	1	11.1
Sample origin		
Endoscopic biopsy	6	66.7
Surgical specimen	3	33.3
Samples		
Tumor	1	11.1
Normal mucosa	2	22.2
Tumor and normal mucosa	6	66.7
Kind of neoadjuvant therapy		
None	4	44.4
Neoadjuvant chemoradiation	4	44.4
Perioperative chemotherapy	1	11.1
pT category		
pT1	1	11.1
pT2	3	33.3
PT3	2	22.2
pT4	1	11.1
No information^a^	2	22.2
pN category		
pN0	3	33.3
pN1	2	22.2
pN2	0	0
pN3	2	22.2
No information^a^	2	22.2
Grading		
G1	0	0
G2	5	55.6
G3	2	22.2
No information	2	22.2

^a^ No further information available since one patient had progression after primary staging and lost to follow‐up in another case.

### 3.2 Workflow

We developed a protocol for isolating the different cell populations (immune cells, fibroblasts, epithelial cells) from EAC and corresponding esophageal mucosa to sequence the transcriptome separately for each cell type. In brief, the workflow consists of a gentle single‐cell dissection, immunofluorescence staining, consecutive flow cytometry sorting, and RNA analysis of each cell fraction (Fig. 1).

**Fig. 2:**
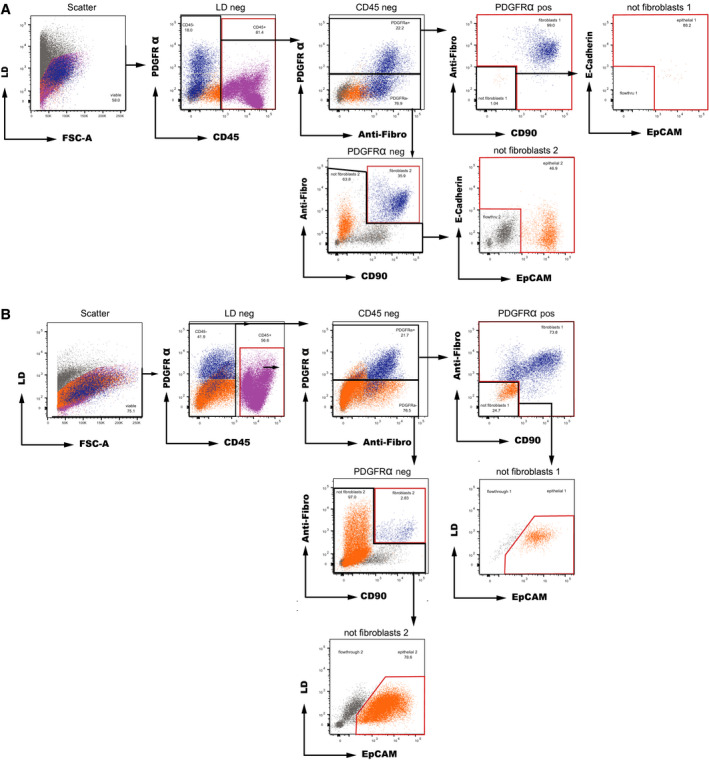
Representative sample processing for separation of EAC cell populations from endoscopic biopsies by flow cytometry sorting with E‐Cadherin co‐staining (A) and without E‐Cadherin co‐staining (B). After initial viability gating, cells were separated into the (a) CD45+ immune cell population (pink); (b) fibroblast cell population (blue), defined as positive for at least two out of three of PDGFRα, anti‐fibroblast, and CD90; and (c) epithelial cell population (dark orange) that were positive for EpCAM and/or E‐Cadherin (A) or simply positive for EpCAM (B). The sorted cell subpopulations are highlighted in red.

Single‐cell suspension was obtained simultaneously by enzymatic dissociation using DNAse I, collagenase IV, and dispase II and simultaneous mechanical dissociation with a gentleMACS™ Dissociator. Antibody staining and subsequent sorting of the single‐cell suspension were implemented on literature‐based antigen selection (see below). An example of flow cytometry sorting strategy is shown in Fig. 2.

For sorting the immune cell population, we selected membrane antigen CD45, a glycoprotein which is expressed on nearly all hematopoietic cells except for mature erythrocytes and platelets (Nakano *et al.*, 1990). CD45 has been revealed as a potent marker to differentiate hematopoietic cells from carcinoma cells in solid and fluid tumor tissue via flow cytometry (Acosta *et al.*, 2016) and serves as a pan‐leukocyte marker (Ruffell *et al.*, 2012), therefore ideal for detection of immune cells.

Epithelial cells were sorted for epithelial cell adhesion molecule (EpCAM), which is exclusively displayed in epithelia and epithelial‐derived neoplasms (Patriarca *et al.*, 2012) and expressed in various carcinomas, including EAC (Sun *et al.*, 2018). EpCAM antibodies are commonly used to detect epithelial cells in flow cytometry, for example, for detecting leptomeningeal metastasis of solid tumors in liquor (Milojkovic Kerklaan *et al.*, 2016) or isolating epithelial cells from normal and tumor tissue (Bantikassegn *et al.*, 2015; Sinha and Lowell, 2016).

Fibroblasts represent a heterogeneous cell population and display an inconsistent expression of surface markers (Lynch and Watt, 2018). Here, isolation of fibroblasts was based on detection of CD90 and PDGFRα and binding of an anti‐fibroblast antibody (Miltenyi, exact antigen is unknown). A cell was defined as ‘fibroblastic’ if positive for at least two of the three surface markers. CD90 has previously been used as a surface marker to isolate (cancer‐associated) fibroblasts in gastrointestinal mucosa and ovarian cancer tissue (Gedye *et al.*, 2014; Kisselbach *et al.*, 2009) and also serves as a widely expressed mesenchymal surface marker (Jiang and Rinkevich, 2018). Likewise, PDGFRα is described as a membrane antigen of mesenchymal cells (Houlihan *et al.*, 2012) and supposed to participate in the recruitment of fibroblasts, pericytes, and endothelial cells during wound healing (Horikawa *et al.*, 2015). There is evidence for a successful enrichment of fibroblasts in flow cytometry using PDGFRα as a surface marker (Pallangyo *et al.*, 2015).

A total of 31 cell populations were successfully separated via flow cytometry and passed RNA‐seq for further analysis. Table 2 illustrates all samples which underwent the complete algorithm.

**Table 2:** Origin of all cell populations included for further RNA‐seq.

Patient No.	Sample origin	Successful RNA‐seq of following cell populations after FACS sorting			
		Tumor tissue	Sample labeling	Normal tissue	Sample labeling
1	Endoscopic biopsy	Immune cells Epithelia Fibroblasts	Tu1_immune Tu1_epithel Tu1_fibro	Immune cells	Mu1_immune
2	Endoscopic biopsy	Immune cells Epithelia Fibroblasts	Tu2_immune Tu2_epithel Tu2_fibro	–	–
3	Endoscopic biopsy	Immune cells Epithelia Fibroblasts	Tu3_immune Tu3_epithel Tu3_fibro	Immune cells	Mu3_immune
4	Endoscopic biopsy	Immune cells Epithelia Fibroblasts	Tu4_immune Tu4_epithel Tu4_fibro	Immune cells Epithelia	Mu4_immune Mu4_epithel
5	Endoscopic biopsy	Immune cells Fibroblasts	Tu5_immune Tu5_fibro	–	–
6	Surgical specimen	^a^	^a^	Immune cells Epithelia	Mu6_immune Mu6_epithel
7	Endoscopic biopsy	Immune cells Epithelia Fibroblasts	Tu7_immune Tu7_epithel Tu7_fibro	^a^	^a^
8	Surgical specimen	^a^	^a^	Immune cells Fibroblasts 1 Fibroblasts 2	Mu8_immune Mu8_fibro1 Mu8_fibro2
9	Surgical specimen	Immune cells Epithelia Fibroblasts	Tu9_immune Tu9_epithel Tu9_fibro	Immune cells Fibroblasts	Mu9_immune Mu9_fibro
Total		*N* = 20		*N* = 11	

^a^ No samples taken.

We used gene ontology analysis, PCA, and RCA as well as population‐specific expression of marker genes to demonstrate the successful separation of the three cell types.

### 3.3 Principal component analysis

**Fig. 3:**
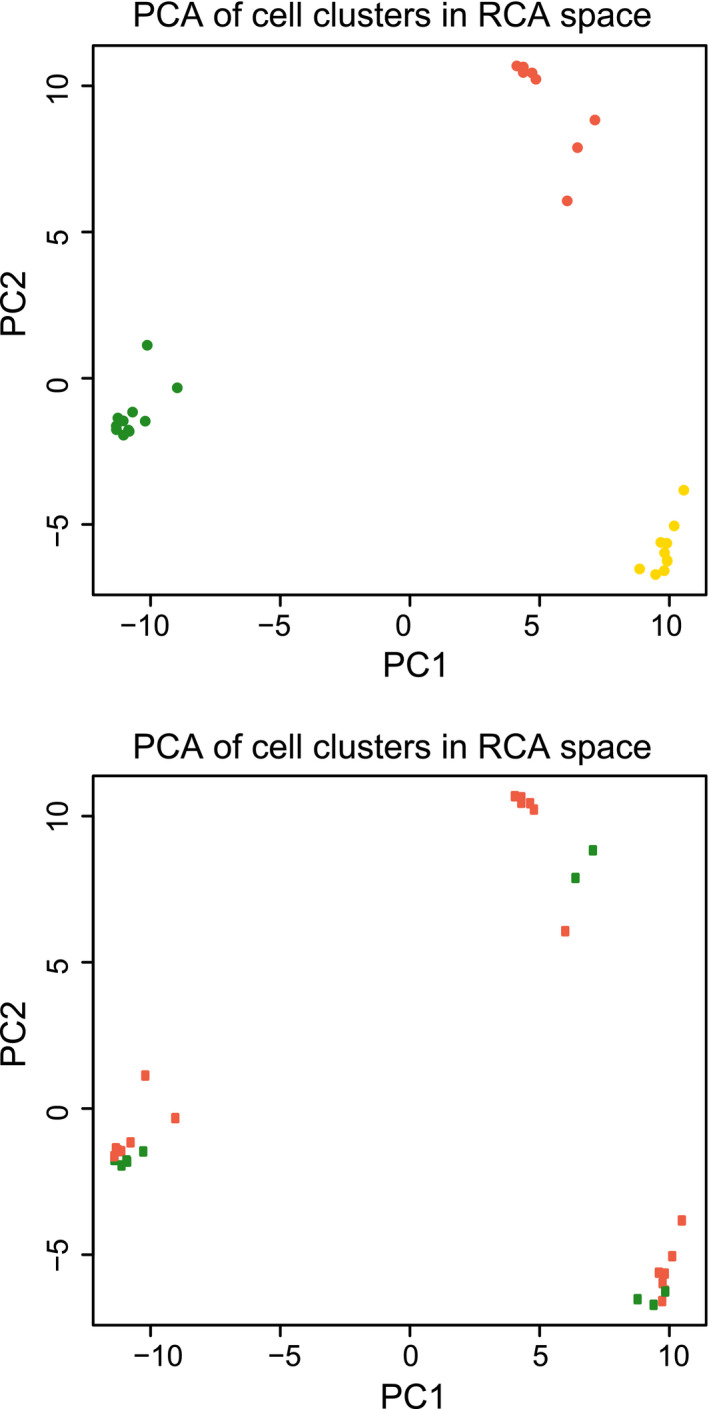
Principal component analysis of RNA‐seq of flow cytometry‐sorted cell fractions. Thirty‐one cell fractions were 3′RNA‐sequenced and plotted by their first two principal components. Top: orange, epithelial cells; yellow, fibroblasts; green, leukocytes. Bottom: orange, tumor cells; green, normal cells.

To confirm an obvious separation of the cell populations, we used dimensionality reduction (PCA) to summarize the data into two dimensions and then visually identify obvious clusters. Cell types sorted by flow cytometry that have similar expression profiles were clustered together. Based on the PCA plot, there were three clean clusters corresponding to the different expected cell types and the first two principal components explain most of the variability (Fig. 3). The PCA supports a successful separation in three different cell populations.

### 3.4 Gene ontology analysis

Gene ontology (GO) is a widely used method to structure a large number of genes in certain categories (GO terms) describing biological processes, molecular functions, or cell components. Based on differential gene expression (Table S2) between the three cell populations, we created a list of the top upregulated genes for each cell type and matched them to specific GO terms of biological processes in which they were significantly overrepresented (FDR < 0.05). In the following, we list the top five GO terms for each sorted cell fraction, and for a complete overview, we refer to the Table S2.

Cells sorted for ‘immune cell population’ showed an enrichment in the categories immune system process, immune response, regulation of immune system process, regulation of immune response, and cell activation, whereas sorted cells of the ‘fibroblast cell population’ were enriched for extracellular matrix organization, anatomical structure morphogenesis, extracellular structure organization, multicellular organism development, and anatomical structure development. Those cells of the ‘epithelial cell population’ had highly expressed genes in GO terms tissue development, epithelium development, epidermis development, epithelial cell differentiation, and cornification.

Gene ontology analysis illustrated expected cell type‐specific GO terms of biological processes for the three sorted cell populations, indicating a successful enrichment of the respective target cell types.

### 3.5 Marker gene analysis

To further confirm the separation of the three cell types, we considered marker genes of our target cell populations and evaluated their distribution within the differently expressed gene lists. Within the immune cell population, there was a significant upregulation of surface proteins CD3 (gene name: *CD3D*), CD4 (*CD4*), CD8 (*CD8A + CD8B*), CD19 (*CD19*), and CD20 (*MS4A1*), all established markers for B/T lymphocytes. Furthermore, monocyte marker CD14 (*CD14*) was significantly upregulated as well as granulocyte marker CD11b (*ITGAM*).

In the fibroblast cell population, there was a significant increase in fibroblast activation protein (*FAP*), commonly described as a marker for (myo)fibroblasts (Shiga *et al.*, 2015). FAP is a surface peptidase with mostly unknown substrates which is involved in numerous physiological processes such as inhibition of fibrinolysis (Hamson *et al.*, 2014). In addition, we observed an upregulation of alpha‐smooth muscle actin/alpha SMA (*ACTA2*) expression, characteristic for fibroblastic cells and which has been widely described in CAFs (Sharon *et al.*, 2013). We further observed in the fibroblast population a significant upregulation of expression of collagen genes (e.g., *COL1A1 and COL3A1*), responsible for the formation of the extracellular matrix (Yue, 2014).

E‐Cadherin (*CDH1*) is a key epithelial marker, responsible for an epithelial barrier between neighboring cells via binding to adjacent cadherins and cytoskeleton (Serrano‐Gomez *et al.*, 2016). *CDH1* was significantly upregulated in the sorted epithelial cells. Similarly, we found a significant increase in expression of epithelial membrane antigen (*EMA*, also known as *MUC1*) in this population. EMA is expressed in various epithelia and responsible for a physical barrier and anti‐adhesive property of the tissue (Nath and Mukherjee, 2014).

In conclusion, cell type‐specific genes showed a significant enrichment in their corresponding target cell populations, supporting the expected cell separation.

### 3.6 Reference component analysis

**Fig. 4:**
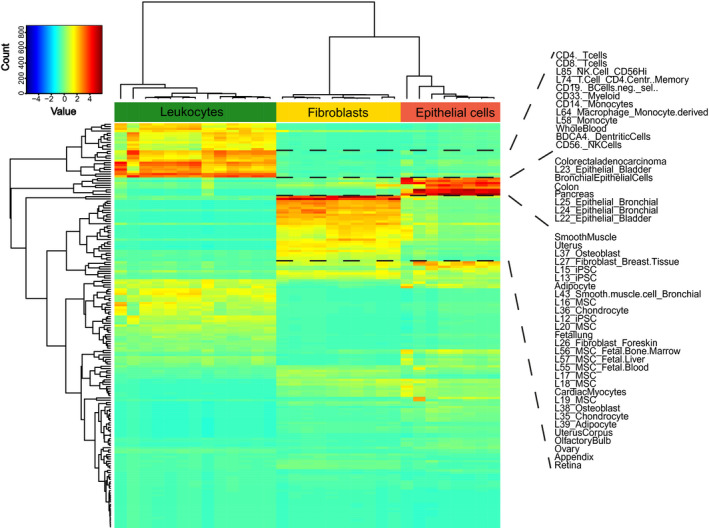
Reference component analysis heat map of the cell populations' transcriptomic profiles correlated with expression profiles of reference tissues and cell lineages. RNA‐seq data of 31 cell fractions plotted in columns with their color‐coded Spearman's correlation values relative to reference expression datasets represented in rows. Cluster color code corresponds to colors in Fig. 3.

Reference component analysis is used to compare gene expression data sets to reference cell lineages. We compared each of the 31 sorted cell fractions by RCA with reference data (Fig. 4). RCA indicated consistent accordance to reference transcriptome data within equally sorted cells. There were three differential mapping (transcriptome correlation) patterns, each belonged to one of the three sorted cell types (‘immune cell population’, ‘fibroblast cell population’, ‘epithelial cell population’), confirming the qualitative sufficiency of the separation process.

Cells sorted as ‘immune cells’ showed a high correlation with reference gene expression of inflammatory cells, for example, natural killer cells, T cells, B cells, monocytes, and macrophages. Cells of the ‘epithelial cell population’ matched to epithelial cell lines of bladder, lung, and colon and non‐specific cells derived from tissue of pancreas and colon. Further correlations were found for trachea and intestinal tissue. Cells sorted as ‘fibroblasts’ revealed correlation with fibroblast cell lines, various mesenchymal stem cell lines, adipocytes, hematopoietic stem cells, endothelial cells, and smooth muscles cells (and organs with high proportion of smooth muscle cells, e.g., uterus). Cancer‐associated fibroblasts (CAFs) have been reported to arise from various origins, for example, normal fibroblasts, adipocytes, bone marrow‐derived cells (including mesenchymal stem cells), and endothelial cells (CD31+) (Wang *et al.*, 2016). Correlations with transcriptome data of these differential cell types might reflect the heterogeneous origin of the fibroblastic cells.

We explored how similar our cell type bulk sequencing was compared with scRNA‐seq and co‐analyzed esophagus epithelial scRNA‐seq data of Owen *et al.* (2018) with epithelial cell types of our analyses. We observed largely congruent correlations with reference transcriptomes (Figs S1 and S2) indicating that the cell type bulk sequencing is pure enough for a representative snapshot of these cells. As expected, smaller cell populations with individual expression profiles can only be seen in scRNA‐seq. Of note, the cell type‐specific sequencing detected more genes per sample compared with scRNA‐seq per cell (average 12 142 vs. 3260 to 4391 genes with ≥ 1 read).

### 3.7 Differently expressed genes between EAC and normal esophageal mucosa

Although the number of tumor–normal pairs does not allow general interpretations, we exploratively compared differentially expressed genes between EAC tissue and normal esophageal mucosa for each sorted cell population (Table S3). We first used GO analysis to categorize generic changes. Only the tumor/normal comparison of the fibroblast cell population showed significant enrichment of GO categories. Interestingly, blood vessel development, angiogenesis, and vasculature development were among the top 10 significantly enriched categories of biological processes (Table S4). These categories remained significant when restricting the analysis to upregulated genes, while downregulated genes were strongly enriched for the establishment of protein localization to endoplasmic reticulum, cotranslational protein targeting to membrane, and regulation of developmental process (Table S4). When focusing on individual genes with reported functions, the fibroblastic population showed strong upregulation of matrix metalloproteinase 11 (*MMP11*) in tumors (adjusted *P* = 1.6 × 10^−15^, Fisher's exact test). MMP11 serves as an endopeptidase‐degrading extracellular matrix (Gómez‐Macías *et al.*, 2018). *MMP11* overexpression in CAFs and cancer cells has previously been described to correlate with an aggressive cancer profile and promotion of metastasis (González de Vega *et al.*, 2019; Peruzzi *et al.*, 2009). Further, an upregulation of glycoprotein *CD38* was detected in the sorted immune cells from EAC tumors (*P* = 8.6 × 10^−7^, Fisher's exact test). CD38, usually expressed on plasma cells and other lymphoid and myeloid cell populations (Morandi *et al.*, 2018), has been revealed to mediate immunosuppression as a tumor escape mechanism, and there is evidence for an unfavorable CD38 influence on tumor progression in esophageal cancer (Chen *et al.*, 2018; Karakasheva *et al.*, 2015).

## 4 Discussion

In an effort to create a scalable approach for cell type‐specific comparison of tumors, we have developed a protocol to dissociate fresh biopsies of EACs and corresponding esophageal mucosa, and sort leukocytes (CD45+), epithelial cells (EpCAM+), and fibroblasts (positive for at least two out of PDGFRα, CD90, anti‐fibroblast), followed by 3′RNA sequencing. This workflow allows us to investigate the transcriptomic changes in the three cell populations in EAC when compared to normal esophageal mucosa. Importantly, this approach is significantly more economic and scalable for investigating transcriptomes of different cell types compared with single‐cell RNA sequencing (scRNA‐seq) and can therefore serve as a cost‐effective alternative to broaden understanding of tumor biology in EAC. In our local setting, the costs for the analysis of a tumor/normal pair with three cell types each by scRNA‐seq (hashing for tumor and normal) would be 3.2 times higher compared with cell type‐specific RNA‐seq.

In order to prove the successful workflow of this approach, we demonstrate a clear separation of our three target cell populations (fibroblasts, immune cells, and epithelial cells) and performed 3′RNA sequencing of these sorted cell types. We used different strategies to verify the identity of the cells. GO analysis elucidated functionally compatible and expected biological processes for the respective cell populations. We provide further evidence for a successful separation by comparing transcriptomic profiles with reference expression data using RCA and obtained three different expression correlation patterns, each specific for the respective target population: Cells sorted for epithelial cell compartment showed high correlation with epithelial cell lineages, the immune cell compartment with reference data of differential lymphocytes, monocytes, and granulocytes and fibroblasts with fibroblasts, various mesenchymal cell lines, and cell lineages that can be considered plausible to serve as origins of CAFs. Literature‐based marker gene analysis within differentially expressed genes further supported a qualitative separation into the three cell compartments.

We focused on optimizing the protocol for small amounts of tissue, allowing to obtain the samples endoscopically at primary staging. Up‐scaling of our protocol will enable us to compare biopsies of treatment‐naïve EAC tissue with normal esophageal mucosa of patient cohorts at moderate cost. Such systematic cell type‐specific analysis of treatment‐naïve, endoscopically obtained samples and correlation with clinical features will allow to find new cell type‐specific pathway alterations, responsible, for example, for treatment response or relapse.

Although we show a potential application of our protocol for biopsies and surgical specimens in normal esophageal mucosa and EAC tissue, the protocol can be further improved. Especially, freezing of dissociated single‐cell suspensions, necessary due to processing logistics, was responsible for a substantial loss of living cells. A continuous workflow without freezing will improve the cell viability and quantity of RNA for sequencing. Since the different samples have varying proportions of the three cell populations, we were not able to obtain sufficient cells for all sorted cell populations. This may explain the lack of significantly enriched GO categories for the leukocyte and epithelial compartments. An increase in the number of viable cells by a continuous workflow will enhance the chance to robustly obtain sufficient amounts of RNA for subsequent 3′RNA‐seq.

In order to demonstrate the potential utility of our approach, we finally compared differently expressed genes between normal esophageal mucosa and EAC tissue and highlighted cell type‐specific alterations. For instance, *CD38* upregulation in immune cells of EAC could establish a possible target of immunotherapy, as suggested in combination with anti‐PD‐L1 therapy (Chen *et al.*, 2018; Mittal *et al.*, 2018). For *MMP11*, which is usually expressed both in epithelial cells and fibroblasts, we found an upregulation restricted to the fibroblast cell population as described earlier (Pedersen *et al.*, 2009). Although sample numbers used here are limited, it is interesting to note that fibroblasts in the tumor showed upregulation of angiogenesis genes, when compared to normal mucosa. Angiogenesis‐promoting properties of CAFs have been described earlier (reviewed in Wang *et al.*, 2019) and are an area of intense investigation in the search for new angles of intervention. Future implementation of the protocol in a large cohort of patients can therefore help to identify targets for novel therapy concepts and complement our understanding of tumor biology in EAC.

In conclusion, we present a new approach for a cost‐effective and scalable procedure to determine cell type‐specific transcriptome alterations in treatment‐naïve EAC tissue with the potential to obtain treatment‐relevant findings in large cohorts of patients with EAC.

## Author contributions

MK, PSP, AQ, HS, S‐HC, and AMH designed the study. MK, PSP, KF‐D, MT, IG‐M, CW, SB, JW, MF, and JA carried out the experiments. PSP, HS, WS, CJB, HA, and S‐HC collected and selected the patients' samples. HL, AQ, and RB performed the histopathological examinations. OVC performed bioinformatic analyses. MK, PSP, OVC, CW, AQ, and AMH interpreted the data. MK, PSP, OVC, and AMH wrote the manuscript with contributions of CW and KF‐D and approval from all authors.

## Conflict of interest

MK is supported by the Koeln Fortune Program/Faculty of Medicine, University of Cologne, grant number 410/2018. PSP is fellow of the Else Kröner Forschungskolleg Cologne ‘Clonal Evolution in Cancer’ (2016‐Kolleg‐19). All other authors declare no conflict of interest.

## Supporting information


**Fig. S1.** Joint normal esophageal mucosa RCA of scRNA‐seq and cell type RNA‐seq.

**Fig. S2.** Normal esophageal mucosa RCA of pseudo‐bulk scRNA‐seq.

**Table S1.** Complete lists of DEGs in a pairwise manner.

**Table S2.** List of the top five Gene Ontology terms for each sorted cell fraction.

**Table S3.** Differentially expressed genes between EAC tissue and normal esophageal mucosa for each sorted cell population (Gene Ontology Analysis).

**Table S4.** Top ten significantly enriched categories of biological processes between EAC tissue and normal esophageal mucosa for each sorted cell population (Gene Ontology Analysis).

## Data Availability

The RNA‐seq data have been deposited at the European Genome‐Phenome Archive and can be accessed via the accession number EGAS00001004053.
