# Supplementary material for: Cell type‐specific transcriptomics of esophageal adenocarcinoma as a scalable alternative for single cell transcriptomics
Source: Mol Oncol. 2020 Apr 21;14(6):1170–84. doi: 10.1002/1878-0261.12680 (PMC7266280; doi:10.1002/1878-0261.12680)
Supplement: Supplementary file 1 — Fig. S1. Joint normal esophageal mucosa RCA of scRNA‐seq and cell type RNA‐seq. [file MOL2-14-1170-s001.pdf]

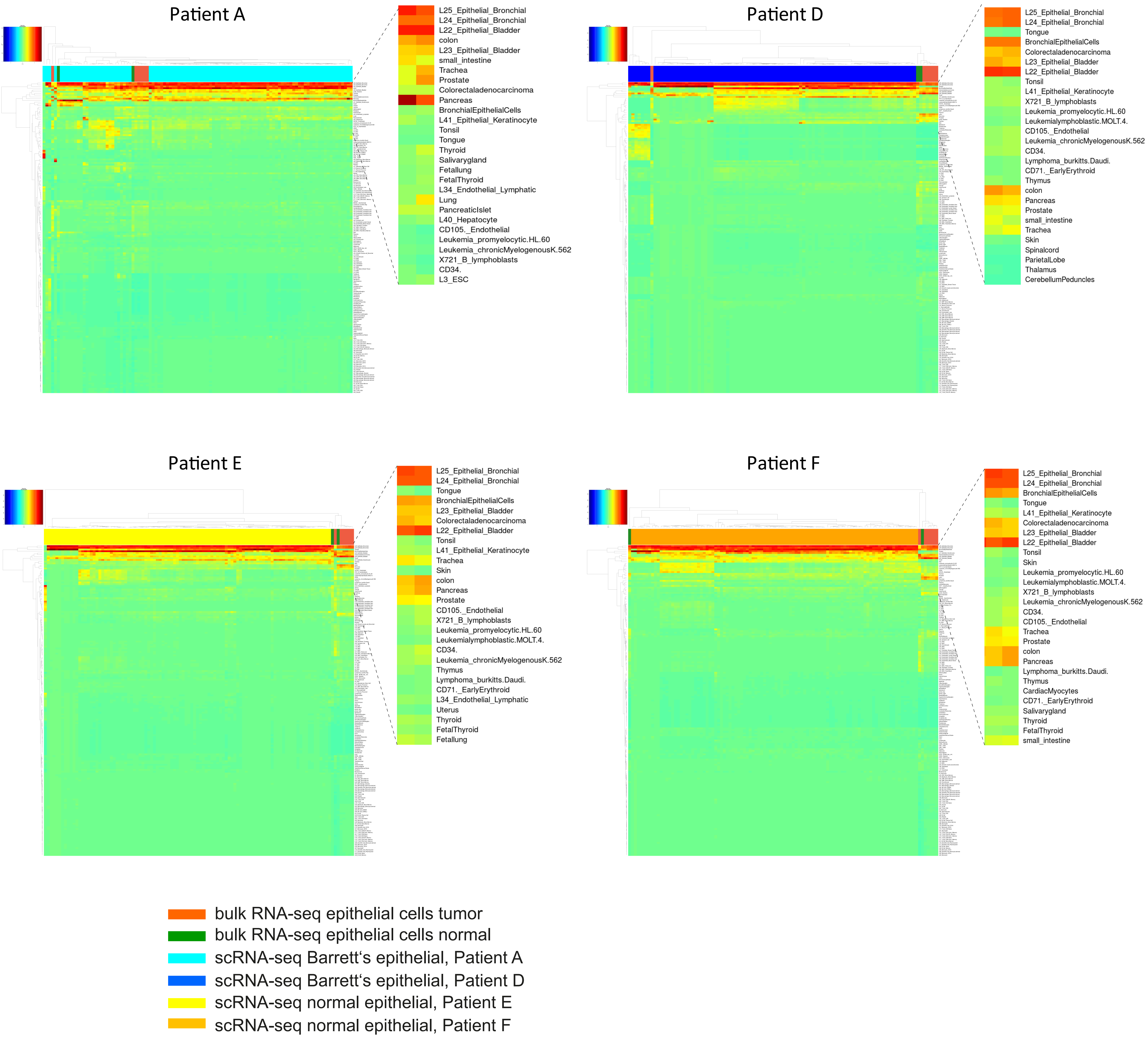

Supplementary Figure 1. Comparison of scRNA-seq and cell type-specific RNA-seq of non-cancer epithelial esophagus cells. Random samples of 100 single-cells each from four different patients with Barrett's esophagus (A and D) or without Barrett's esophagus (E and F) were selected from (Owen et al., 2018) and a matrix of counts was generated by adding the raw counts from the bulk RNA-seq of epithelial cells from flow-sorted samples. RCA clustering shows high projection scores onto epithelial tissues for both, single-cells as well for FACS-sorted samples.
