# Supplementary figures and images for: Cell type‐specific transcriptomics of esophageal adenocarcinoma as a scalable alternative for single cell transcriptomics
Source: Mol Oncol. 2020 Apr 21;14(6):1170–84. doi: 10.1002/1878-0261.12680 (PMC7266280; doi:10.1002/1878-0261.12680)

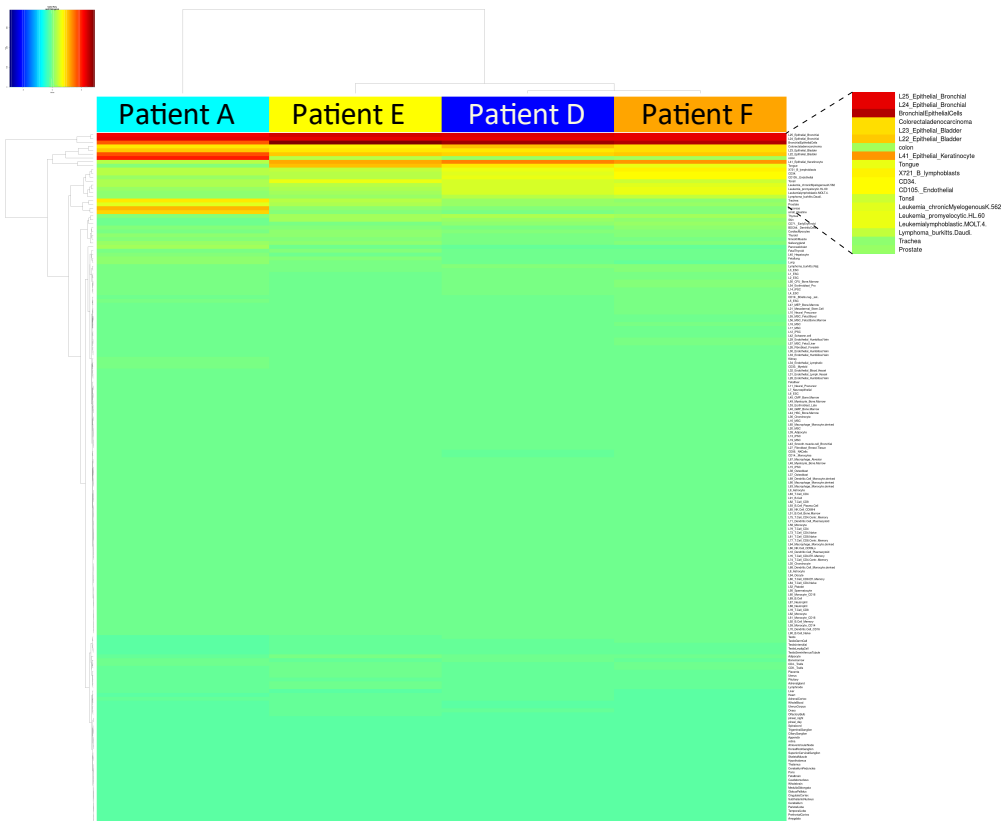

Supplement: Supplementary file 2 — Fig. S2. Normal esophageal mucosa RCA of pseudo‐bulk scRNA‐seq. [file MOL2-14-1170-s002.pdf]
